# First person – Mark Khoury

**DOI:** 10.1242/bio.059499

**Published:** 2022-07-25

## Abstract

First Person is a series of interviews with the first authors of a selection of papers published in Biology Open, helping early-career researchers promote themselves alongside their papers. Mark Khoury is first author on ‘ Minimal functional domains of the core polarity regulator Dlg’, published in BiO. Mark is a PhD student in the lab of Dr David Bilder at University of California, Berkeley, Berkeley, CA, USA, investigating the mechanisms that establish and maintain epithelial cell polarity.


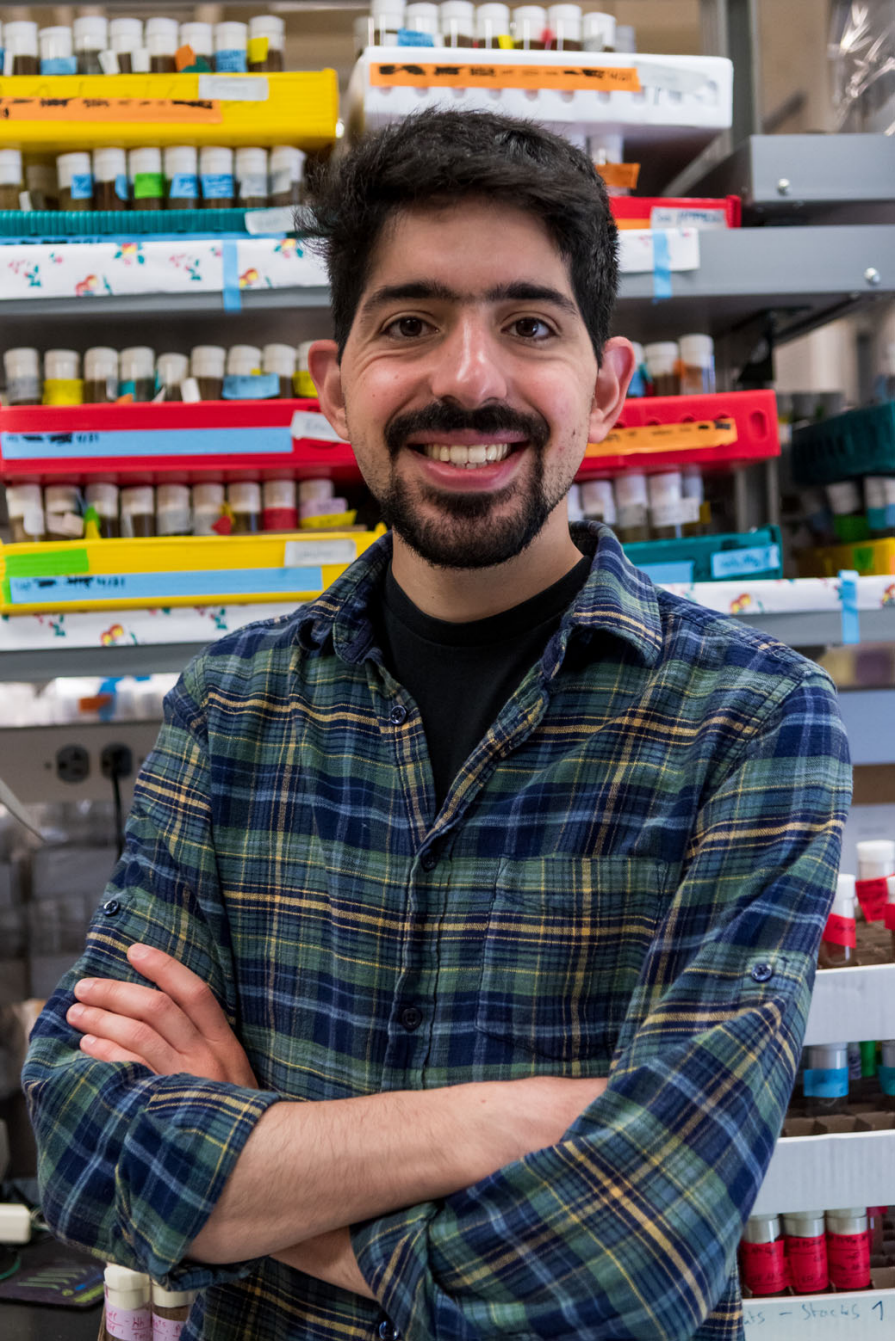


**Mark Khoury**

**What is your scientific background and the general focus of your lab?**

I've had varied scientific interests that have led me to my current work on epithelial developmental and cell biology. As an undergraduate, my very first lab experience was in Dr Lydia Contreras' lab working on RNA and synthetic biology. This was an important foundation because I learned all my basic molecular biology skills during this time. I was actually a neuroscience major though, and I was very interested in neurodegenerative disorders. I then joined Dr Eric Klann's lab and ended up working on the cellular basis of neurodevelopmental disorders, which, although not my initial interest, was a great experience because I was given a lot of independence to shape my own project. However, in my senior year at NYU, I took a developmental biology class and fell in love with development and the amazing things cells do in embryos, especially the spatial regulation of morphogenesis and patterning. This was a pivotal moment in my scientific journey and motivated me to pursue my PhD with Dr David Bilder, studying how epithelial cells polarize. In the Bilder lab, we study epithelial cell biology, and our major interests include mechanisms of epithelial polarity, morphogenesis of epithelial organs and epithelial tumorigenesis and tumor-host interactions. We use *Drosophila* as a model for this work, which is fantastic because flies offer such a wide range of genetic tools and accessible tissue types.

**Figure:**
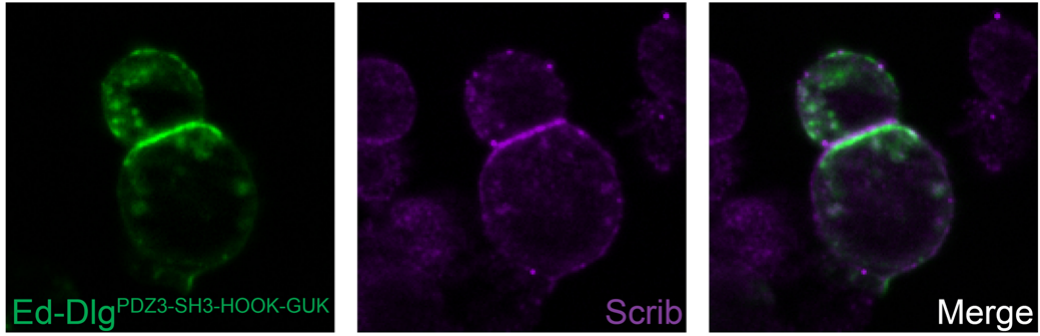
**A cell adhesion-based synthetic cell polarity system, adapted from Johnston et al. (2009), enables *in vitro* polarization of Dlg in cultured cells.** This recapitulates the *in vivo* ability of Dlg to recruit Scrib and provides a powerful system to dissect the underlying molecular mechanisms.

**How would you explain the main findings of your paper to non-scientific family and friends?**

All the cells in your body must have a proper sense of direction and be able to distinguish their tops from their bottoms in order to do their specific jobs. This is called polarity, and allows them to hold onto their neighbor cells, crawl in specific directions and transport nutrients, among other things. In epithelial cells, which are the cells that make up the lining of most of your organs, we know that specific genes and proteins are required for them to have this top-bottom directional sense. In particular, we know that a group of three proteins called ‘the Scrib module’ is important for defining the bottom sides of epithelial cells, although we don't know exactly how they do this. In this paper, we set out to understand how the members of the Scrib module – Scrib, Dlg and Lgl – work together to define the bottom sides of cells. Our results led us to focus on Dlg. Dlg is a large protein with many parts, which has made it difficult to understand what all these parts are doing and how their activities are coordinated. To understand how Dlg works, we cut up the protein into many small pieces to find out what each part, or combination of parts, does. We found that there are two specific, small parts of Dlg that are most important for its activity in epithelial polarity. This allowed us to define a ‘minimal’ set of parts of Dlg that are alone sufficient to function. This was surprising given how many parts Dlg has in total and suggests that the other parts may only be required in certain contexts. Based on these findings, we made hypotheses about how Dlg could be interacting with the other Scrib module proteins to establish epithelial polarity and incorporated these ideas into a model for how the bottom side of epithelial cells is defined in contrast to the top side.

“Our work makes progress towards a more complete understanding of the molecular functions of the Scrib module proteins and contributes to future comprehensive models of basolateral polarity establishment.”

**What are the potential implications of these results for your field of research?**

By defining the minimal components of the Dlg protein that are required and sufficient to support epithelial polarity, our work makes progress towards a more complete understanding of the molecular functions of the Scrib module proteins and contributes to future comprehensive models of basolateral polarity establishment. Additionally, our work advances knowledge of the relationships between Scrib, Dlg and Lgl at the basolateral cortex. These are both understudied areas with many unknowns, and our work provides a basis for future studies that will hopefully uncover the more detailed mechanisms of how these proteins function.

**What has surprised you the most while conducting your research?**

One thing that surprised me early on is how different the answers can be from the same question, depending on what model system you use. A lot of my results have been negative or different from what's been seen in the vertebrate literature, which was initially confusing and concerning to me. Now, I think it highlights the importance of using diverse systems to answer basic biological questions, because it turns out the answers are often context dependent. It also has taught me the importance of reading broadly and not just focusing on papers from within your own model organism community.

**What, in your opinion, are some of the greatest achievements in your field and how has this influenced your research?**

I am always amazed by the elegant mechanistic studies on the apical polarity regulators, the Par complex. The level of detail that these works have uncovered about how these essential proteins function, and the clever combinations of genetics and biochemistry they use to solve these problems is really impressive. In particular, I love re-reading the three papers that described the phosphorylation-dependent mechanism that segregates Par-3 from Par-6 and aPKC in *Drosophila* and began to differentiate the specific functions of the apical Par proteins (Par-3, Par-6 and aPKC) in epithelia (Morais-de-Sá et al., 2010; Walther and Pichaud, 2010; Krahn et al., 2010); I find them so elegant, and each time I read them I'm surprised by new details I didn't notice before. They were super insightful and important for the field. In our work, we've been inspired by these types of approaches and by work from the *C. elegans* polarity field, so I've tried to channel that kind of creativity in our own investigations of the molecular mechanisms of Scrib module function.

**What changes do you think could improve the professional lives of early-career scientists?**

I think there are many systemic issues, but one that I'm particularly invested in is improving active mentoring. What I mean by ‘active’ is mentorship that goes beyond passive advising and is tailored to individual trainees' needs. Active mentoring is dynamic and requires bidirectional communication. For example, instead of editing and re-writing a trainee's manuscript draft for them, an active mentor points out specific places where changes should be made and helps the trainee understand how to improve the writing and why the changes benefit the impact of the writing. Trainee success is generally linked to, and dependent on, our direct advisors and their mentorship. I have been very fortunate to have had excellent mentors who were actively invested in my training, but unfortunately this is not the case for everyone. Focused, active mentoring allows trainees to reach their full potential and increases the likelihood that they are successful. Focusing on more active mentoring by advisors would help develop more well-rounded trainees and would benefit the entire community in the long run because these trainees can then continue the culture of active mentoring. I've been particularly inspired by my PhD advisor David Bilder, who is a very strong, active mentor, to advocate for and cultivate a culture of strong, equitable mentorship as I move forward in my career, because everyone deserves the best chance to succeed.

**What's next for you?**

I just graduated from UC Berkeley in May and will be starting a postdoc with Dr Ya-Chieh Hsu at Harvard this summer.
